# Can scoliosis follow up by surface topography (Biomod-L®) securely predict Cobb angle progression? Longitudnal study; preliminary results on 60 patients

**DOI:** 10.1186/1748-7161-7-S1-O20

**Published:** 2012-01-27

**Authors:** M De Seze, G De Korvin

**Affiliations:** 1 University Hospital Bordeaux Cedex, France; 2 Priv Prm Practicesaint Gregoire, France

## Background

The gold standard parameter for scoliosis follow-up is the Cobb angle from full spine radiographs. However, the repetition of X-rays on children and adolescents may increase future cancer risks [1,2]. Our project is to space out X-rays assessments by using a Moiré based Surface Topography device (Biomod-L®).

Two reference postures have been selected after a preliminary study: 1) Joined elbows and coiled shoulders (dorsal hump measurement); 2) Erected position, hands grasping wall bars (all other measurements).

## Purpose

Can the progression of Biomod-L® parameters securely predict the progression of Cobb angles measured on X-rays?

## Materials and methods

60 patients (mean age 13,4 years old ; 9-18) who had undergone at least two simultaneous X-Rays + Biomod-L® assessments were included in a row. This provided a total of 75 “follow up segments” distributed on different periods of growth, preliminary follow up and treatment follow up.

The X-rays criteria were +3° for progression and -5° for improvement. The Biomod-L® progression was assessed on the hump, lordosis, spinal curves and list measurements, and on a subjective comparison of the fringe mapping.

## Results

For worsening prediction: sensitivity 90%, negative predictive value 90%, specificity 60%, positive predictive value 59%. For improving prediction: sensitivity 50%, negative predictive value 87%, specificity 91%, positive predictive value 62%.

## Conclusion

According to the sensitivity and negative predictive value for worsening prediction, Biomod-L® seems a reasonably liable tool for detecting slight progressions of the Cobb angle and to be used as a trigger for X-Rays controls.
